# Natural Phenolic Compounds for the Control of Oxidation, Bacterial Spoilage, and Foodborne Pathogens in Meat

**DOI:** 10.3390/foods9060794

**Published:** 2020-06-16

**Authors:** Aphrodite I. Kalogianni, Thomai Lazou, Ioannis Bossis, Athanasios I. Gelasakis

**Affiliations:** 1 Laboratory of Anatomy and Physiology of Farm Animals, Department of Animal Science, Agricultural University of Athens (AUA), Iera Odos 75 str., 11855 Athens, Greece; afrokalo@aua.gr (A.I.K.); bossisi@aua.gr (I.B.); 2 Laboratory of Hygiene of Foods of Animal Origin—Veterinary Public Health, School of Veterinary Medicine, Faculty of Health Sciences, Aristotle University of Thessaloniki, 54124 Thessaloniki, Greece; tlazou@vet.auth.gr

**Keywords:** natural phenolic compounds, antioxidant activity, antimicrobial activity, meat, meat-based products, foodborne pathogens, spoilage

## Abstract

Alternative technologies for long-term preservation, quality assurance, and safety of meat are continuously pursued by the food industry to satisfy the demands of modern consumers for nutritious and healthy meat-based products. Naturally occurring phenolic compounds are considered promising substances by the meat industry for their antioxidant and antimicrobial properties, while consumers seem to embrace them for their claimed health benefits. Despite the numerous in vitro and in situ studies demonstrating their beneficial effects against meat oxidation, spoilage, and foodborne pathogens, wide application and commercialization has not been yet achieved. Major obstacles are still the scarcity of legislative framework, the large variety of meat-based products and targeted pathogens, the limited number of case-specific application protocols and the questionable universal efficiency of the applied ones. The objectives of the present review are (i) to summarize the current knowledge about the applications of naturally occurring phenols in meat and meat-based products, emphasizing the mechanisms, determinants, and spectrum of their antioxidant and antimicrobial activity; (ii) to present state-of-the-art technologies utilized for the application of phenolic compounds in meat systems; and (iii) to discuss relevant regulation, limitations, perspectives, and future challenges for their mass industrial use.

## 1. Introduction

During the last 70 years, an increased demand for foods of animal origin and especially meat and meat products has been observed worldwide. This has resulted in the intensification of livestock production and the development of globalized logistics and complex transboundary trade of meat products. In addition, evolution of nutritional habits, changes in lifestyle, and other societal, religious, and monetary factors have reshaped the meat industry and its future growth and direction. Despite the continuous emergence of new food trends, meat and meat products will remain a significant source of animal-derived protein and essential amino acids in human nutrition [1].

To meet consumer demands for fresh meat and adhere to food safety regulations, extensive supply chains have been developed worldwide utilizing cold chain logistics. Despite the universal adoption of basic food cold chains, alternative technologies for long-term preservation, quality assurance and meat safety are continuously pursued. Meat and its products are ideal substrates for the growth and propagation of spoilage microorganisms and common foodborne pathogens, rendering them as high-risk perishable foods with potential public health implications [2,3]. More than 20% of worldwide meat production, which is equivalent to 75 million slaughtered cows, is lost or wasted along the food supply chain due to spoilage [4]. In monetary terms, meat losses account for ~4% of total food losses and cost about $150 billion USD, representing more than 20% of the global economic cost due to food losses [5].

Meat contamination with pathogens may originate from any point across the “farm to fork” continuum, which includes animal farming, transportation, slaughtering, processing, packaging, distribution, and meal preparation in the household environment [2,6]. The basic pillar for meat hygiene is the control measures to eliminate or reduce pathogenic or spoilage microorganisms in farms, slaughterhouses, and meat processing plants. These measures are integrated in the hazard analysis and critical control point (HACCP) plan, which is a legislative requirement in developed countries [3,7,8,9]. Currently, hygiene and preservation methods utilized by the meat industry include (i) heat treatment (e.g., scalding of carcasses, pasteurization, water, and steam boiling treatment); (ii) refrigeration (e.g., chilling, freezing, blast chilling, superchilling); (iii) high hydrostatic pressure (HHP); (iv) packaging (vacuum, modified atmosphere, and active packaging); (v) ionizing radiation; (vi) chemical preservatives (carbon dioxide, chlorine dioxide, ozone, lactoferrin, organic acids (e.g., lactic and citric acid), salts (e.g., nitrates, sodium lactate, sodium chloride, sodium benzoate, trisodium phosphate, potassium sorbate)), and bioactive compounds (e.g., natural phenolic compounds, nisin, pentosin, chitosan, lysozyme); and (vii) hurdle technologies (i.e., a combination of existing and novel food preservation techniques). Variability in the effectiveness and applicability of the aforementioned methods, the need for further optimization/validation in some of them, intellectual property rights (IPRs), alterations in organoleptic traits of the product, health concerns (real or perceived) such as the possible carcinogenic effects of nitrates [10], and consumers’ skepticism are driving the decision-making process of the meat industry toward their exploitation on an evidentiary case-by-case basis [3,7,11,12].

To meet the ever-growing skepticism of the consumer, biocontrol and natural additive compounds for the prevention of meat oxidation, spoilage, and foodborne pathogens have emerged as novel preservation technologies. They exploit the antioxidant and antimicrobial properties of bacteriophage viruses and biomolecules (e.g., bacteriocins, natural organic acids, peptides and other groups of organic compounds) produced by lactic acid bacteria, plants, and animals. These compounds are used as biopreservatives according to the type (raw or cooked meat), specific conditions, (storing temperature, pH, etc.), and targeted pathogens in meat and meat byproducts [3,7,13]. Among these compounds, naturally occurring phenols are considered promising substances against meat spoilage and foodborne pathogens. They are plant-derived biomolecules with proven industrial and consumer acceptance for their antioxidant and antimicrobial capabilities [14,15].

The objectives of the present review are (i) to summarize the current knowledge about the applications of naturally occurring phenols in fresh meat, meat products, minced meat, and meat preparations, as defined in Regulation (EC) No 853/2004 [16], emphasizing on the mechanisms, determinants and spectrum of their antioxidant and antimicrobial activity, and (ii) to present state-of-the-art technologies utilized for the application of phenolic compounds in meat systems, and (iii) to discuss relevant regulation, limitations, perspectives, and future challenges for their mass industrial use.

## 2. Meat Oxidation, Spoilage, and Foodborne Pathogens

Oxidation of lipids and proteins in meat affect both its quality and safety. In addition to the organoleptic (flavor, aroma and color) and nutritional deterioration (denaturation of fatty acids and proteins), oxidation reduces the shelf-life of meat and derived products and undermines their safety due to production of toxic substances [15,17]. Oxidation is favored by the presence of polyunsaturated fatty acids in meat [17,18]. Double bonds in polyunsaturated fatty acids function as ideal initiators for the oxidation process reacting with atmospheric oxygen and other fatty acids and leading to the formation of hydroperoxides and free radicals [15]. The process continues until the final products, such as aldehydes, ketones, and hexanes, cannot further support the oxidation cycle [18]. In turn, the products of lipid oxidation and free radicals promote protein oxidation results in protein carbonylation, polymerization, and coagulation [19]. These chemical changes decrease protein solubility and prevent natural proteolysis, which adversely affects meat organoleptic traits, such as tenderness and juiciness [17,20]. Apart from the lipid and protein profile and content, the oxidation process is further influenced by other factors such as heat, light, metal ions, heme pigments, low pH, and oxidative enzymes [17,21].

Several foodborne illnesses linked to the consumption of contaminated, insufficiently cooked, or inadequately preserved meat and meat-based products are reported globally, undermining public health and causing significant monetary losses for the meat industry [3,7,22]. Incidence of foodborne illnesses associated with the consumption of meat and products thereof, apart from those attributed to major foodborne outbreaks, is likely to be underestimated, as corresponding cases remain underdiagnosed or unreported, particularly in countries with inefficient or inexistent monitoring programs [6,23,24]. Additionally, lipid and protein oxidation have been linked to cytotoxicity, neurotoxicity, mutagenicity and carcinogenesis [19,25]. In particular, the end-products of protein oxidation may constitute risk factors for cancer and diabetes [19], while the aldehyde groups originating from lipid oxidation have been linked to metabolic disorders and other diseases of the kidneys, the vasculature and the colon [26].

During the last decade, the annual cases of foodborne illnesses in humans in the EU have fluctuated without intense variation over the years [27]. In general, meat and meat products along with eggs and egg products are the top two most frequently reported food vehicles of animal origin pathogens associated with foodborne outbreaks in the EU [27]. Similarly, each year in the United States of America, meat and products thereof are responsible approximately for 30% of total foodborne outbreaks and 50% of foodborne illness cases, and they represent the most commonly reported food vehicle of animal origin for the pathogens implicated in the corresponding human cases [28,29]. The main agents causing meat borne outbreaks in the EU and in the USA are *Salmonella* spp.; bacterial toxins produced by *Bacillus* spp., *Staphylococcus* spp., *Clostridium* spp. (other than *Clostridium botulinum*), and other unspecified bacterial toxins; *Campylobacter* spp., *Trichinella* spp., norovirus, and other caliciviruses; *Cl. Botulinum*; and other bacterial agents (such as *Aeromonas hydrophila*, enterotoxigenic *Escherichia coli* (ETEC), *Enterococcus*, *Shigella* spp., *Yersinia enterocolitica*, Shiga toxin-producing *E. coli* (STEC), and *Listeria* spp.) [27,29].

## 3. Natural Phenolic Compounds

Natural phenolic compounds are biomolecules with at least one aromatic ring linked to hydroxyl substituents, and they are derived as secondary metabolites of plant tissues [14,30,31,32]. They contribute to the appearance, taste and basic functions of tissues and provide innate defensive functions in many plant species. Their classification is complex and can be based on their carbon chain length, the side groups of the aromatic ring, their distribution in nature, and the part of the plant they derive from [33]. Based on the molecular structure of their aromatic ring, they are classified into simple phenols and benzoquinones (C_6_), phenolic acids (C_6_-C_1_), acetophenones and phenylacetic acids (C_6_-C_2_), hydroxycinnamic acids, phenylpropenes, coumarins-isocoumarins and chromones (C_6_-C_3_), naphthoquinones (C_6_-C_4_), xanthones (C_6_-C_1_-C_6_), stilbenes and anthraquinones (C_6_-C_2_-C_6_), flavonoids (C_6_-C_3_-C_6_), lignans and neolignans (C_6_-C_3_)_2_, and lignins (C_6_-C_3_)_n_ [33]. Natural phenolic compounds are abundant in plants and can be found in herbs, spices, vegetables, fruits, wine, essential oils, olive oil, and oil seeds [17,18]. Although they are non-nutritional components, they are utilized by the food industry for their antioxidant, antimicrobial, antifungal and antiviral functions [34].

Natural phenolic compounds with useful applications in food industry are (i) phenolic acids with one aromatic ring, further subdivided into the hydroxybenzoic acids (e.g., gallic, protocatechuic, vanillic, and syringic acid) and the hydroxycinnamic acids (e.g., p-coumaric, caffeic, and ferulic acid); the antioxidant activity of these compounds is related to the number of hydroxyl groups in their molecule; (ii) flavonoids (e.g., flavanols, flavones, flavanones, flavan-3-ols, isoflavones, anthocyanins), which constitute the largest subcategory in natural phenolics and their structure consists of a 15-carbon skeleton arranged in two phenyl rings and a heterocyclic ring; (iii) quinones made of two carbonyls (e.g., benzoquinones, anthraquinones); (iv) tannins, which are abundant in fruits and are formed by quinones or flavonoids; (v) coumarins with a combination of benzene and alpha-pyrone ring, which are potentially toxic but also useful for their antimicrobial activity if used properly; (vi) lignans; (vii) stilbenes; and (viii) curcuminoids [32,34,35,36]. Essential or volatile oils from plants have characteristic aromas and are composed of a mixture of several phenolic compounds which serve as the basic active ingredients (terpenes, terpenoids, and phenylpropanoids). Typical examples of essential oils rich in phenolic compounds that have been used in the food industry are prepared from oregano (carvacrol, thymol, p-cymene, γ-terpinene), clove (eugenol), coriander (linalool), ginger (α-pinene, cineole, borneol, geraniol, α-curcumene, camphene and eucalyptol), rosemary (carnosic acid, carnosol, rosmadial, genkwanin, rosmarinic acid, 1,8-cineole, α-pinene, limonene and camphor), sage (α-thujone, β-thujone, camphor, 1,8-cineole, borneol, viridiflorol), thyme (thymol, carvacrol, ρ-cymene, γ-terpinene, linalool), and mint (menthol) [34,37,38,39].

### 3.1. Antioxidant Capacity of Phenolic Compounds

Antioxidant substances prevent or hamper the oxidation chain reaction by capturing free radicals, reducing oxygen, deactivating singlet oxygen (^1^O)_2_, conjugating the metal ions, eliminating the hydroperoxides, and absorbing UV light [15,17,18]. Phenolic compounds are considered effective antioxidants due to their ability to deactivate and stabilize free radicals by incorporating them into their aromatic ring [17,18,21,40] and to absorb UV light [18]. The phenolic subgroup of flavonoids can also act as metal chelator, mainly for Fe^3+,^ inhibiting the oxidation process [18,21]. Contrary to synthetic phenolic antioxidant substances, which have been used for decades in the food industry and are accused for toxicity and carcinogenesis [41,42,43], natural phenolic compounds have beneficial effects on human health by protecting against oxidative-stress-related chronic diseases [40,44,45,46]. Several studies have investigated the antioxidant capacity of phenolic compounds in meat and its organoleptic traits. Table 1 presents applications of antioxidant phenolic compounds in meat-based products, the effects of phenolic compounds on lipid and protein oxidation, and on product organoleptic traits (color, flavor, and taste). In addition, it summarizes the comparisons between phenolic compounds and conventional food additives regarding their antioxidant. Phenolic compounds are referred to only in cases where analytical laboratory techniques have been used to detect them.

### 3.2. Antimicrobial Activity of Phenolic Compounds

#### 3.2.1. Antimicrobial Activity Mechanisms 

Many phenolic compounds serve as efficient antimicrobial agents [34,37,90]. The mechanisms of their antimicrobial properties have not been completely clarified as yet; however, the prevalent hypothesis is that they do so by destabilizing the microbial cell surface and cytoplasmic membranes [37,91,92,93,94]. This may lead to irreversible damage of the cell wall and various intracellular organelles, coagulation of cell compartments, and inhibition of intracellular enzymes. In particular, the hydrophobic phenolic compounds are bound by the lipid bilayer of the microbial cell membrane causing its structural disruption and loss of integrity [95,96], leading to the creation of pores, the flow of intracellular components in the extracellular space, and the functional dysregulation of proteins such as the Na^+^/K^+^-ATPase pump [96,97,98]. In addition, the phenolic ring, due to its hydroxyl group and the indispensable double bonds, has the capacity to act as a transmembrane transporter of cations, causing influx of H^+^, efflux of K^+^, and suppression of ATP synthesis [99,100]. Phenolic compounds may interact with intracellular components and DNA after disruption of the cell wall and entry into the cell [95,100]. The destruction of intracellular membranes causes release of free radicals that in turn can lead to DNA damage and lipid oxidation. As an adaptive response to this phenolic “attack,” microbes modify their gene expression to reduce aerobic metabolism and increase production of antioxidant and DNA repair enzymes [101,102]. Suppression of aerobic metabolism by itself restricts microbe motility and biofilm formation, which are conditions that favor survival.

#### 3.2.2. Antimicrobial Activity Associated Factors

The antimicrobial activity of natural phenolic compounds mainly depends on their chemical structure and concentration. Several additional factors, however, have been identified that could limit the antimicrobial activity of the phenolic compounds when applied in meat systems. The antimicrobial activity is adversely affected by increased pH; low water activity; and salt, fat, and complex carbohydrate content [34,91,103,104,105,106]. Reduced pH and increased concentration of phenolic substances enhance the hydrophobicity of essential oils favoring their attachment to the pathogen’s lipid cell membranes and therefore, their antimicrobial activity [104,105,107]. In general, the hydrophobic nature of phenolic compounds facilitates their accumulation in fat [108]. The high concentration of fat in meat does not favor the contact between phenolic compounds and the pathogenic microorganisms that accumulate in the hydrophilic phase of meat [91,106]. Proteins create strong complexes with phenolic compounds, and this may reduce their antioxidant and antimicrobial capacity. However, the results from relative studies are contradictory, and the effect of proteins on antimicrobial or antioxidant activity of phenolic compounds is not clarified [109]. Finally, high temperature can eliminate the antimicrobial activity of natural phenolic compounds particularly during packaging [34], while low storage temperature favors it [106,108].

Synergistic and antagonistic interactions contribute to the antimicrobial activity of phenolic compounds; however, the mechanisms have not been yet elucidated. These interactions are observed when phenolic substances are used in combination or in cases of essential oils which contain more than one phenolic compound [39]. The fortified or reduced antimicrobial activity of essential oils compared to that of individual phenolic compounds reveals the synergistic or antagonistic interactions, respectively [39,97,110]. For example, carvacrol (the main phenolic compound of oregano oil) and a fraction of dill oil with d-limonene and carvone when compared with crude essential oils demonstrated increased antimicrobial capacity, indicating that there is an antagonistic interaction between some of their components [39,111]. Examples of synergistic action have been documented in the following combinations of phenolic compounds: (i) carvacrol, thymol, and eugenol against *Listeria innocua*; (ii) carvacrol and p-cymene against *Bacillus cereus*; (iii) cinnamaldehyde and eugenol against *Staphylococcus* spp., *Micrococcus* spp., *Bacillus* spp., and *Enterobacter* spp. [39,97]; (iv) carvacrol and thymol against *E. coli* O157:H7, *Staphylococcus aureus*, *L. innocua*, *Saccharomyces cerevisiae*, and *Aspergillus niger* [34]; (v) geraniol and menthol against *S. aureus*; (vi) thymol and menthol against *B. cereus* [112]; and (vii) cranberry (ellagic acid) and oregano (rosmarinic acid) extract against *Listeria monocytogenes* [104,113]. Synergistic interactions between phenolic compounds and antibiotics against foodborne pathogens have also been observed. For example, the combination of green tea extract with oxacillin is effective against *S. aureus* [114]; gallic acid with amikacin, norfloxacin, gentamicin, and sulfamethoxazole [115] or catechin and ciprofloxacin [116] against *E. coli*; p-coumaric acid, sinapic acid, caffeic acid, vanillic acid, gallic acid, and taxifolin with ciprofloxacin and erythromycin against *Campylobacter jejuni* [117]; and ellagic and tannic acids with ovobiocin, coumermycin, chlorobiocin, rifampicin, and fusidic acid against *Acinetobacter baumannii* [118]. The aforementioned synergistic interactions may indicate that one phenolic compound facilitates the antimicrobial function of another compound or antibiotic and vice versa; e.g., the phenolic compound initiates cell membrane rupture facilitating the entry of an antibiotic compound [97].

#### 3.2.3. Antimicrobial Activity Spectrum

##### In Vitro Antimicrobial Activity of Phenolic Compounds

Several in vitro studies have demonstrated the antimicrobial properties of natural phenolic compounds against foodborne pathogens. Regardless of their chemical structure, phenolic compounds act primarily against Gram-positive and to a lesser extent on Gram-negative bacteria (Table 2). The list of Gram-positive bacteria inhibited by various essential oils and spices is remarkable (see Table 2).

Gram-negative bacteria are less sensitive to the antimicrobial activity of phenolic compounds [91,106,121,133] due to the hydrophilic outer membrane of lipopolysaccharides that hinders entry of lipophilic phenolic molecules [95]. Nevertheless, phenolic compounds from berries (anthocyanins, flavonols and hydroxy-cinnamates) have proved to be somewhat active against *Salmonella* spp. (Gram negative) but not against Gram positive bacteria [134]. Also, a citrus oil called Brazilian orange terpenes, has presented higher antibacterial action against *E. coli* (Gram negative) than *Lactobacillus rhamnosus* (Gram positive) [135].

##### In Situ Antimicrobial Activity of Phenolic Compounds in Meat Systems

Various studies have been conducted in situ for the verification of antimicrobial activity of phenolic compounds in meat-based products. In these studies, phenolic compounds were used mainly in the form of essential oils and plant extracts and their efficiency against a wide range of pathogens was assessed under various combinations of substances, concentrations, and types of meat. Successful applications of several natural antimicrobials containing phenolic compounds are shown in Table 3.

## 4. Application of Phenolic Compounds in Meat

### 4.1. Direct Application in Meat

Additives containing phenolic compounds can be either incorporated directly into meat-based products or in bio-based functional packaging materials. Herbs and spices or their extracted oils can be added as such, thus exploiting their distinct flavor [34,103,167]. The most appropriate application of herbs and spices on meat products depends on the phenolic compounds of the extracted essential oils and their antimicrobial properties (e.g., targeted microorganisms) [34]. Effectiveness depends on the type of application and could even vary in the same or similar meat products [168].

Active food packaging using bio-based materials that also incorporate phenolic compounds is an emerging technology. The packaging material in these cases could protect meat against oxidation and spoilage [3,17,34,169,170]. Currently, active packaging includes sachet-based applications gradually releasing phenolic compounds, pads with incorporated phenolic compounds and in direct contact with the product, edible or polyethylene bioactive films coated with essential oils [34,73,169,171,172,173]. Techniques for the incorporation of phenolic compounds into packaging films is complex and needs to account for factors such as heat and pressure applied during processing and packaging. These factors can adversely affect the molecular structure and therefore the functionality of the incorporated phenolic compounds [174]. During the manufacturing of active packaging materials, microencapsulation of phenolic compounds is currently exploited to protect their molecular structure and secure their antioxidant and antimicrobial activity [73,175]. State-of-the-art nanotechnologies that have been exploited in food nano-encapsulation applications include (i) biopolymer and lipid-based nanoparticles and cyclodextrins; (ii) nano-emulsions; (iii) nano-spray drying; and (iv) electro-spinning [174,175,176,177,178]. These technologies facilitate the carriage and delivery of phenolic compounds, protecting them from challenging conditions within food (e.g., pH, temperature, and other organic compounds). Additionally, they improve their solubility and bioavailability, and mitigate the risk of developing undesirable flavors [175,179,180].

### 4.2. Incorporation in Animal Diets

The addition of phenolic compounds in animal diets has been used to improve the antioxidant capacity of the produced meat. In ruminants, natural phenolic compounds have been related with (i) inhibitory effect on fibrolytic bacteria and protozoa, (ii) reinforcement of rumen bypass of polyunsaturated fatty acids and protein metabolism, and (iii) the production of conjugated linoleic acid [181,182,183] contributing to the qualitative improvement of the derived meat products. In monogastric animals, phenolic compounds have been found to act beneficially for the intestinal microbiota and the stability of polyunsaturated fatty acids in the intestine and muscle tissues [184]. For example, in broilers, incorporation of polyphenols extracted from the industrial waste of olive oil processing in their diets improved the antioxidant capability of their meat [185]. Similarly, the addition of gallic and linoleic acid to broiler diets had beneficial effects on their lipid metabolism, their productivity, the nutritional value and quality of meat, and its antioxidant and antimicrobial capacity [186], whereas oregano and laurel oil improved growth rate and reduced lipid oxidation [187]. Feeding rosemary extract in turkeys had a beneficial effect against lipid oxidation and spoilage of their meat [188]. In goats, consumption of tannin-rich leaves from woody plants improved the oxidative stability of the derived meat due to modulation of the fatty acid profile [189]. In rabbits and lambs, consumption of chestnut tannins increased their immune response under stress conditions, and improved growth rate, meat quality and antioxidant capacity [190,191]. In pig diets, incorporation of wood extract and oregano oil increased the presence of antioxidant enzymes in animals, prevented lipid oxidation and improved meat color [192]. Similarly, the addition of rosemary extract in lamb diets delayed lipid oxidation, color deterioration, and bacterial spoilage of the produced carcass [193], whereas tannins and oregano oil improved antioxidant status and color of meat [194,195]. Hence, the improvement of meat antioxidant and antimicrobial capacity results both from the direct accumulation of the phenolic compounds in meat and their indirect activity, which reinforces the health and welfare status of animals, therefore mitigating the oxidative stress and the respective degradation of their products.

## 5. Regulation, Limitations, and Challenges in the Use of Phenolic Compounds

Health risk assessment of phenolic compounds (essential oils, extracts and purified compounds) is necessary prerequisite for their commercial use in food products. This means that the inclusion of phenolic compounds in food legislation and their subsequent approval for commercial use by the meat industry, demand scientific evidence and the conduction of relevant risk assessment considering human health. This implies that the safety, health, and quality claims of phenolic compounds when applied in meat systems should be documented; records available by the European Food Safety Authority and corresponding committees of other countries need to be considered as well. This process is cost-intensive and time-consuming particularly when purified phenolic compounds are to be used [97], limiting their industrial application.

To date, the European Commission has approved (EU, 1333/2008 and 1129/2011) the application of rosemary extract (E392) as a food additive for meat products to concentration up to 150 mg/kg [38,196,197] and several other phenolic compounds (linalool, thymol, eugenol, carvone, cinnamaldehyde, vanillin, carvacrol, citral, and limonene) in the list of food flavoring substances, as described in the Regulation (EU) No 872/2012 [198]. Rosemary extract has been also approved for use in food industry in Japan, China [196], the US, and recently in Australia and New Zealand (Australia New Zealand Food Standards Code—Standard 1.3.1—Food Additives). The use of phenolic compounds in active food packaging (e.g., edible films, encapsulation) according to the Regulation (EU) No 450/2009 demands their incorporation in the list of approved food additives, given that they come into contact with food [199]. Likewise, the Food and Drug Administration (FDA) in the US has included several essential oils containing phenolic compounds such as clove, oregano, thyme, nutmeg, basil, mustard, cinnamon oil, and even estragole (prohibited in EU as genotoxic) in the list of Generally Recognized as Safe (GRAS) substances [39,97]. Hence, the commercial use of phenolic compounds in meat-based products to exploit their antioxidant and anti-spoilage activity is currently scarce.

Phenolic compounds naturally occurring in fruits and vegetables or produced by microorganisms could modify food flavor [90]. Several extracts and essential oils from fruits and vegetables (e.g., citrus, carrots, potatoes, orange, oregano) give sweet, sour, bitter, or astringent flavor due to their phenolic content, which precludes their unconditional use in meat preparations and meat products [7,15,91,106,156,165]. Adoption of the recommended concentrations, addition of modulatory substances like salt [91] and application of appropriate technologies, like micro-encapsulation of phenolic compounds, are significant measures to mitigate undesirable effects of phenolic compounds on organoleptic traits of meat-based products [15,180].

The interaction of phenolic compounds with meat components (lipids, proteins, carbohydrates) and their effect on intrinsic quality traits of meat (e.g., pH, organoleptic attributes) limits their wide application [37,39]. Although phenolic compounds are considered to decrease the digestibility of carbohydrates and proteins, inhibit the bioavailability of amino acids and bind the available iron, these effects have not been observed in meat [90,109]. In any case, further in situ experiments are necessary to evaluate the activity and possible anti-nutritional effects of natural phenolic compounds in meat-based products under various conditions.

Phenolic compounds are commonly added in meat in the form of essential oils. However, the intrinsic composition of each essential oil and its interrelated antimicrobial capacity depend mainly on the plant species and also, on the season, the age and the origin of the plant, environmental factors that can affect its phenotypic expression and the used extraction process (e.g., solvent extraction, fermentation, distillation, effleurage) [97,149,200,201,202]. Also, the source of natural phenolic compounds determines their purity and, therefore, their antimicrobial capability [123]. Finally, the cost for the extraction of natural phenolic compounds is affected by the degree of purification and the improvement of extract quality by removing potentially toxic organic components [203]. Protocols to standardize the composition of essential oils and the extraction of phenolic compounds are critical for the evaluation of their inhibitory activity and the cost-effectiveness of their application in meat-based products. Another way to increase the content of phenolic compounds in meat is via the incorporation of phenolic compounds in the diets of farm animals. This is a growing research field, currently challenged by knowledge gaps regarding the determination of the most effective phenolic compounds (type and concentration) and the most appropriate techniques to incorporate them in farm animal diets, without adversely affecting animal productivity, health, and welfare status.

In general, the demand for nutritious, functional, healthy and safe meat-based products is growing in modern societies, motivating the meat industry to develop new technologies. Among these technologies, the ones based on the addition of natural compounds such as fatty acids, minerals, vitamins, natural antioxidants, dietary fibers, probiotics or bioactive peptides are emerging [204,205,206,207]. Except for the antioxidant and anti-spoilage function of natural phenolic compounds, they also act beneficially for human health [208]. For example, flavonols have presented an anti-obesity effect [68,209], a protective effect against oxidative stress-induced neurotoxicity [210], and anti-diabetic [211] and potential anti-tumor activity [212]. Some natural phenolic compounds (e.g., date palm extract) have been found to exhibit an inhibitory function against the invasion and metastasis of cancer cells [45,213]. Another interesting aspect for public health is the fact that their antimicrobial action renders them promising alternatives to chemical additives and synthetic antimicrobials and, thereby, useful tools against the development of antibiotic resistance.

## 6. Conclusions

Despite the numerous in vitro and in situ studies demonstrating the beneficial effects of natural phenolic compounds against meat oxidation, spoilage, and foodborne pathogens, wide application and commercialization in the meat industry has not been yet achieved. Major obstacles are still the scarcity of a legislative framework, the large variety of meat-based products and targeted pathogens, and, in many cases, the limited number of case-specific application protocols and the questionable efficiency of those applied. The immediate establishment of a legislative framework for the use and assessment of natural phenolic compounds in meat-based products is a crucial prerequisite towards their massive application in meat industry and for the consumers’ acceptability. Regardless of direct or indirect applications of phenolic compounds in meat systems, their effectiveness is neither universally accepted nor unquestionable. Instead, it is defined in a case-by-case manner, taking into account the phenolic compound itself, the meat-based product and the targeted microorganisms. In any case, real-world, in situ testing of these compounds is of paramount importance to verify their functionality and significance for the meat industry. Nano-encapsulation of the phenolic compounds in active packaging systems is a state-of-the-art technology with, though, limited in-depth studies regarding possible adverse effects on human health and the environment. Also, natural phenolic compounds and other natural antioxidant and antimicrobial substances could be jointly tested for the production of novel commercial food additives that will facilitate meat quality and safety assurance. Furthermore, the existence of synergistic activity between natural phenolic compounds and other preservation methods (e.g., high pressure processing) need to be investigated to achieve the optimum outcomes with the minimum effects on the organoleptic traits of meat. In the future natural phenolic compounds are expected to constitute an innovative tool integrated into meat systems to satisfy the ever-increasing demands for natural, quality, safe, and healthy food.

## Figures and Tables

**Table 1:** Antioxidant effect of phenolic compounds in meat and meat-based products.

Meat/Meat-Based Product	Treatment (Main Phenolic Compound)	LA ^1^	PA ^2^	COL ^3^	FL/TA ^4^	FA ^5^	Ref ^6^
	**Pork meat and meat-based products**						
Fresh meat	Lotus extract (tannins, flavonoids)	++	N/A	++	0/++	N/A	[47]
Foal steaks	Oregano essential oil (N/A) *	++	++	+	++	N/A	[48]
	Green tea extract (N/A) *	++	++	0	0		
Patties	Rosemary extract (N/A)	++	+	0	N/A	AA (-)	[49]
	Green tea extract (N/A)	++	+	0	N/A	AA (-)	
	Green tea extract (N/A)	++	N/A	++	N/A		[50]
	Grape extract (N/A)	++	N/A	-	N/A	BHT (-)	
	*Cudrania tricuspidata* leaf powder (flavonoids)	++	N/A	++	N/A	N/A	[51]
	Black currant (*Ribes nigrum* L.) extract (anthocyanins)	++	++	+	0	N/A	[52]
	Licorice (*Glycyrrhiza glabra*) extract [hispaglabridin (A and B), glabridin,4′-O-methylglabridin, isoprenylchalcone, liquiritigenin, isoliquir-itigenin, formononetin]	++	N/A	N/A	+	BHA (0)	[53]
	Tea polyphenols (catechins) *	++	N/A	++	++	N/A	[54]
	Rosemary extract (N/A)	++	++	+	0	BHT (-)	[55]
	Lemon balm extract (N/A)	++	++	+	0	BHT (-)	
	Grape seed extract (N/A)	++	N/A	0	0	N/A	[56]
	Bearberry extract (N/A)	++	N/A	0	0	N/A	
	Berries extract (N/A)	++	N/A	N/A	N/A	N/A	[57]
Meatballs	*Ginkgo biloba* leaves extract (polyphenols, phenolic acids, flavonols)	++	N/A	N/A	N/A	BHT (-)	[58]
Liver pâté	Chestnut extract (N/A)	++	N/A	N/A	0	BHT (+)	[59]
	Grape extract (N/A)	++	N/A	N/A	0	BHT (-)	
	Tea extract (N/A)	++	N/A	N/A	0	BHT (-)	
	Date palm by-products (N/A)	++	0	-	0	N/A	[60]
	Date palm paste & annatto extract (N/A)	+	N/A	N/A	N/A	N/A	[61]
Sausages	Lutein (lutein)	0/+	N/A	0	0	N/A	[62]
	Seasamol (seasamol)	++	N/A	0	0	N/A	
	Ellagic acid (ellargic acid)	++	N/A	0	-	N/A	
	Olive leaf extract (Oleuropein, verbascoside, luteolin-	++	N/A	0	0	N/A	
	7-O-glucoside, apigenin-7-O-glucoside, tyrosol, hydroxytyrosol)						
	Grape seed extract (N/A)	++	N/A	+	++	BHT (-)	[63]
	Chestnut extract (N/A)	++	N/A	+	++	BHT (-)	
	Adzuki bean extract (N/A)	++	N/A	-/0	0	BHT (0)	[64]
	Jaboticaba peel extract (N/A)	++	N/A	0	0	N/A	[65]
	Green tea extract (N/A)	++	-	-	0	N/A	[66]
	Rosemary extra (N/A)	++	-	-	-	N/A	
	Sage (N/A)	++	+	++	0	N/A	[67]
	Lotus seed epicarp extract (flavonoids)	++	N/A	N/A	N/A	N/A	[68]
	Shiitake powder (N/A)	++	N/A	0	+	SN (-)	[69]
	Banana male flowers extract (flavonoids)	++	N/A	0	0	N/A	[70]
Bacon	Tea polyphenols (N/A)	++	N/A	N/A	N/A	TC (-)	[71]
Ham	Garlic, cinnamon, clove and rosemary essential oils (N/A)	++	++	++	N/A	SC-SE [LA (-), PA (0)]	[72]
	*Rosa canina* L. extract (N/A)	++	++	0	N/A	SC-SE [LA (0), PA (-)]	
	**Beef meat and meat-based products**						
Fresh meat	Rosemary extract (carnosic acid, carnosol)	++	N/A	N/A	N/A	BHT (-), PG (+)	[73]
	Polyvinylpolypyrrolidone brewery washing solution (benzoic acid derivatives, flavan-3-ols, cinnamic acids, flavanones, flavones, flavonols, acetophenone derivates, stilbenoids) *	++	N/A	N/A	N/A	BHT (-),PG (0)	
	Lotus rhizome knot and lotus leaf extracts (tannins and flavonoids)	++	N/A	++	0/++	N/A	[47]
	Olive hydroxytyrosol or 4-dihydroxyphenylglycol (hydroxytyrosol, 4-dihydroxyphenylglycol) *	++	N/A	N/A	N/A	N/A	[74]
Patties	Chamnamul (*Pimpinella brachycarpa*) extract (N/A)	++	N/A	++	N/A	BHT (+)	[75]
	Fatsia (*Aralia elata*) extract (N/A)	++	N/A	++	N/A	BHT (0)	
	Chestnut extract (N/A)	++	N/A	+	0	BHT	[76]
	Seasonings derived from wine pomace (N/A)	++	N/A	N/A	N/A	Sulfites (-)	[77]
Meatballs	Film with sage (*Salvia officinalis*) (N/A) *	++	N/A	0	-	N/A	[78]
	Film with *Laurus nobilis* (N/A) *	++	N/A	0	-	N/A	
	Pomegranate peel extract (N/A)	++	++	+	0	BHT (-)	[79]
Sausages	Grape seed extract (N/A)	++	N/A	+	+	AA, PG (-)	[80]
	Green tea extract (N/A)	++	N/A	+	0	N/A	[81]
	Stinging nettle extract (N/A)	++	N/A	-	0	N/A	
	Olive leaves extract (N/A)	++	N/A	-	0	N/A	
	**Poultry meat and meat-based products**						
Fresh meat	Pequi (*Caryocar brasiliense*) waste extract (phenolic acids, flavonoids, anthocyanins)	++	++	-	N/A	BHT (-)	[82]
	Jucara (*Euterpe edulis*) waste extract (phenolic acids, flavonoids, anthocyanins)	0	++	-	N/A	BHT (+)	
Meat wafer	Apple peel (N/A)	++	N/A	++	++	N/A	[83]
	Banana peel (N/A)	++	N/A	++	++	N/A	
	Aloe vera gel (N/A)	++	N/A	++	++	N/A	
	Drumstick leaf powder (N/A)	++	N/A	++	++	N/A	
Patties	Pomegranate juice, pomegranate rind powder extract (N/A)	++	N/A	0	0	BHT (-)	[84]
	**Lamb meat and meat-based products**						
Patties	Tomato by-products extract (N/A)	0	0	0	N/A	SA (0)	[85]
	Red grape by-products extract (N/A)	++	++	0	N/A	SA (-)	
	Olive by-products extract (N/A)	++	++	0	N/A	SA (-)	
	Pomegranate by-products extract (N/A)	0	0	0	N/A	SA (0)	
Burger	*Origanum vulgare* extract (rosmarinic acid, cathechin/ epicatechin derivative, 4-(3,4-Dihydroxybenzoyloxymethyl)phenyl-β-D-glucopyranoside, naringenin)	++	++	+	0	SE (-)	[86]
Sausages	*Origanum vulgare* extract (N/A)	++	++	-	0/-	SE (0)	[87]
	**Mixed meat sausages**						
Sucuk	Green tea extract (N/A)	++	N/A	0	++	BHT (-)	[88]
(lamb- beef)	*Thymbra spicata* oil (N/A)	++	N/A	0	++	BHT (-)	
Poultry- pork	Nutmeg essential oil (N/A)	++	N/A	0	++	N/A	[89]

^1^ lipid antioxidant effect, ^2^ protein antioxidant effect, ^3^ color effect, ^4^ flavor/taste effect, ^5^ food additives other than phenolic compound (in this column, the brackets indicate equal (0), increased (+) or decreased (-) antioxidant capacity of food additives compared to phenolic compounds), ^6^ references. * application of phenolic treatment in packaging. +: poor positive effect, ++: strong positive effect, -: negative effect, 0: no effect (columns LA, PA, COL, and FL/TA; the grading system reflects the opinion/conclusions of the corresponding referenced citation). AA: ascorbic acid, BHT: butylated hydroxytoluene, BHA: butylated hydroxyanisole, SN: sodium nitrite, TC: α-tocopherol, SC-SE: sodium citrate- sodium erythorbate, PG: propyl gallate, SA: sodium ascorbate, N/A: relative data is not available.

**Table 2:** In vitro antimicrobial activity of phenolic compounds or fractions.

Phenolic Compound	Targeted Microorganisms	Ref ^1^
**Phenolic acids**		
p-coumaric acid	*Saccharomyces cerevisiae*, *Escherichia coli*, *Salmonella enterica* serovar Typhimurium, MRSA, *Staphylococcus aureus*, *Bacillus subtilis*, *Shigella dysenteriae*, *Streptococcus pneumoniae*	[90,100,119]
Ferulic acid	*S. cerevisiae*, *Lactobacillus plantarum*, *S. aureus*, *Staphylococcus epidermidis*, MRSA	[90,119]
Caffeic acid	*L. plantarum*, *E. coli*, *S. aureus*, *S. epidermidis*, MRSA, *Serratia marcescens*, *Proteus mirabilis*	[90,119,120]
Gallic acid	*E. coli*, *S. aureus*. *Klebsiella pneumoniae*	[120,121]
Vanillic cid	*E. coli*, *S. aureus*, MRSA, *P. mirabilis*, *K. pneumonia*, *Candida albicans*, *C. neoformans*	[119,120,122]
Protocatechuic acid	*E. coli*, *S. aureus*, *L. monocytogenes*, *Streptococcus agalactiae*	[119,120]
Syringic acid	MRSA, *L. monocytogenes*	[119]
2,4-dihydroxybenzoic acid	*E. coli*, MRSA, *Enterococcus faecalis*	[119]
**Flavonoids**		
Epicatechin	*E. coli*, *E. coli* O157:H7, *S. enterica* serovar Choleraesuis, *Salmonella enterica* serovar Enteritidis, *Salmonella enterica* serovar Paratyphi	[123]
Epigallocatechin	*E. coli*, *Salmonella* spp., *S. aureus*, *Vibrio* spp.	[124]
Εpigallocatechin-3-O-gallate	*E. coli*, *Salmonella* spp., *S. aureus*, *Vibrio* spp.	[124]
Procyanidins	*S. aureus*	[124]
Theaflavins	*S. aureus*, *Vibrio* spp.	[124]
Prodelphinidin	*E. coli*, *Salmonella* spp., *S. aureus*, *Vibrio* spp.	
Myricetin	*E. coli*, *E. coli* O157:H7, *S.* Choleraesuis, *S.* Enteritidis, *S.* Paratyphi	[123]
Quercetin	*E. coli*, *E. coli* O157:H7, *S.* Choleraesuis, *S.* Enteritidis, *S.* Paratyphi, *S. marcescens*, *P. mirabilis*, *K. pneumonia*	[123]
		[120]
Rutin	*E. coli*, *E. coli* O157:H7, *S.* Choleraesuis, *S.* Enteritidis, *S.* Paratyphi, *S. marcescens*, *P. mirabilis*, *K. pneumoniae*, *Pseudomonas aeruginosa*, *Acinetobacter baumannii*	[123]
		[120,125]
Xanthohumol	*E. coli*, *E. coli* O157:H7, *S.* Choleraesuis, *S.* Enteritidis, *S.* Paratyphi	[123]
**Quinones**		
Thymoquinone	*E. coli*, *E. coli* O157:H7, *S.* Choleraesuis, *S.* Enteritidis, *S.* Paratyphi	[123]
Hydroquinone	*S. aureus*	[121]
**Tannins**		
Tannins	*L. plantarum*	[126]
Castalagin	*E. coli*, *Salmonella* spp., *S. aureus*, *Vibrio* spp.	[124]
Punicalagin	*S. aureus*, *Vibrio* spp.	[124]
Tannic acid	*S. aureus*, *Vibrio* spp.	[124]
Geraniin	*S. aureus*, *Vibrio* spp.	[124]
**Coumarins**		
Coumarin	*E.coli*, *S.* Typhimurium, *Salmonella enterica* serovar Infantis, *Enterobacter aerogenes*	[127]
**Curcuminoids**		
Curcumin	*E. coli*, *E. coli* O157:H7, *S.* Choleraesuis, *S.* Enteritidis, *S.* Paratyphi	[123]
**Other polyphenols**		
Chlorogenic acid	*E. coli*, *E. coli* O157:H7, *S.* Choleraesuis, *S.* Enteritidis, *S.* Paratyphi	[123]
**Terpenes**		
Carvacrol	*E. coli,* STEC, *S. aureus*, *P. fluorescens*, *Bacillus cereus*	[112,121]
Carvone	*S. aureus*	[112]
Eugenol	*E. coli*, *E. coli* O157:H7, *S.* Choleraesuis, *S.* Enteritidis, *S.* Paratyphi, *S. aureus*, *B. cereus*	[112,123]
Thymol	*E. coli*, *E. coli* O157:H7, STEC, *S.* Choleraesuis, *S.* Enteritidis, *S.* Paratyphi, *S. aureus*, *Pseudomonas fluorescens*, *B. cereus*	[121,123]
**Phenolic fractions**		
*Scrophularia frutescens*	*Bacillus* sp.	[128]
*Ginkgo biloba*	*E. coli*, *S.* Typhimurium, *S. aureus*, *Listeria monocytogenes*, *Listeria innocua*, *Streptococcus pyogenes*, *Shigella dysenteriae*, *E. aerogenes*, *Vibrio vulnificus*	[129]
Oil vegetation water	LAB, *E. coli* O:157 H7, *S.* Typhimurium, *S. aureus*, *S. xylosus*, *L. monocytogenes*, *L. innocua*, *Pseudomonas* spp.	[130]
Olive oil	*Campylobacter jejuni*, *C. coli*	[131]
Garlic	*E. coli*, *S. aureus*	[132]

^1^ references, STEC: Shiga toxin-producing *E. coli*, LAB: lactic acid bacteria, MRSA: Methicillin-resistant *S. aureus*.

**Table 3:** In situ antimicrobial activity of natural phenolic compounds in meat-based products.

Meat/Meat-Based Products	Treatment	Targeted Microorganisms	FA ^1^	Ref ^2^
	**Pork meat and meat-based products**			
Foal steak	Oregano (O) *	TVC, LAB	N/A	[48]
	Green tea (E) *	*Pseudomonas* spp., Enterobacteriaceae, yeasts, molds		
Patties	Green tea (E)	TVC, LAB, psychrotrophic anaerobic bacteria, *Pseudomonas* spp.	BHT (-)	[50]
	Grape (E)			
	Tea polyphenols (P) *	TVC	N/A	[54]
Sausages	Rosemary (E)	Enterobacteriaceae, *Pseudomonas* spp., yeasts, molds	CH(0), TC (-)	[136]
	Olive mill wastewater (E)	Molds	Ethanol (-)	[137]
	Olive vegetation water (E)	*Staphylococcus* spp., molds	N/A	[138]
	Oregano (O)	Aerobic heterotrofic bacteria, *Escherichia coli*	N/A	[139,140]
	Shiitake (P)	TVC	SN (0)	[69]
Liver pâté	Date palm and annatto (E)	TVC	N/A	[61]
	Pomegranate peel (E)	*Listeria monocytogenes*	N/A	[141]
Ham	Carvacrol, cinnamaldehyde *	*L. monocytogenes*	N/A	[142]
Hamburger	Cranberry pomace (E)	LAB, TVC, *L. monocytogenes*, *Brochothrix thermosphacta*, *Pseudomonas putida*	N/A	[143]
Bacon	Tea polyphenols, grape seed (E)	TVC, Enterobacteriaceae, Micrococcaceae, yeasts, molds	TC (-)	[71]
	Gingerol	TVC, Enterobacteriaceae, Micrococcaceae		
	Liquid smoke	*E. coli*, *Salmonella enterica* serovar Choleraesuis, *Staphylococcus aureus*, *L. monocytogenes*	N/A	[144]
Salami	Olive mill wastewater (E)	*L. monocytogenes*	Nitrate (-)	[14]
	**Beef meat and meat-based products**			
Fresh meat	Oregano (O)	*S. aureus*	AC (-)	[145]
	Oregano and cranberry (EP)	*L. monocytogenes*	LA (-)	[104]
	Grape seed (E)	*E. coli* O157:H7, *Salmonella enterica* serovar Typhimurium, *L. monocytogenes*	BHA/BHT (-)	[103]
	Pine bark (E)			
	*Malpighia punicifolia* (E)	*B. thermosphacta*, *Pseudomonas* spp.	N/A	[146]
Minced meat	Sage (O)	TVC, Enterobacteriaceae, *Salmonella enterica* serovar Anatum, *Salmonella enterica* serovar Enteritidis, *S. aureus*, *Bacillus cereus*, yeasts, molds	N/A	[147]
	Prickly pear (E)	TVC, Enterobacteriaceae, *Pseudomonas* spp.	N/A	[148]
	*Pistacia lentiscus* (O) *Satureja montana* (O)	*L. monocytogenes*	N/A	[149]
	*Rumex tingitanus* (E)	*L. monocytogenes*	N/A	[150]
Patties	Thymol	Enterobacteriaceae, Coliforms	N/A	[151]
	Wine pomace seasoning	TVC, LAB	Sulfites (+)	[152]
	Grape pomace (E)	Coliforms, Enterobacteriaceae, yeasts, molds	N/A	[153]
	Chamnamul and fatsia (E)	Coliforms	BHT (0)	[75]
Sucuk	Black carrot concentrate	Yeasts, molds	SN (-)	[154]
	**Poultry meat and meat-based products**			
Fresh meat	Carvacrol, cinnamaldehyde	*E. coli* O157:H7, *S.* Enteritidis	N/A	[142]
	Carvacrol vapor	*S.* Enteritidis,	N/A	[155]
	Oregano (O)	LAB, Enterobacteriaceae, *B. thermosphacta*, Pseudomonas spp., yeasts	N/A	[156]
	Pomegranate (E)	*Salmonella enterica* serovar Kentucky, *S.* Enteritidis	N/A	[157]
	*Zanthoxylum rhetsa* (E) *	TVC, *Staphylococcus* spp., Coliforms	N/A	[158]
	Eugenol *	*Campylobacter jejuni*	AC (-)	[102]
Minced meat	Clove (O) *	*S.* Typhimurium, *L. monocytogenes*	N/A	[159]
Sausages	Rosemary, Chinese mahogany (E)	TVC	N/A	[160]
Salami	Prickly pear (E)	LAB, *Staphylococcus* spp.	SN-CO (-)	[161]
	**Lamb meat and meat-based products**			
Fresh meat	Oregano (O)	TVC, Enterobacteriaceae	N/A	[162]
	Thyme (O)	*Pseudomonas* spp.		
Patties	Tomato by-products (E)	Mesophile and psycrotrophic microorganisms, Enterobacteriaceae		[85]
	Pomegranate by-products (E)		SA (0)	
	Red grape by-products (E)	Mesophile microorganisms		
	Olive by-products (E)		SA (0)	
Minced meat	Oregano (O)	*S.* Enteritidis	Nisin (-)	[163]
	**Mixed meat sausages**			
Beef & pork	β-resorcylic acid, carvacrol, trans-cinnamaldehyde	*L. monocytogenes*	DMSO (-)	[164]
	Cranberry (P)	*L. monocytogenes*	NLD (0)	[165]
	*Kitaibelia vitifolia* (E)	*E. coli*	N/A	[166]
Poultry & pork	Nutmeg essential (O)	TVC	N/A	[89]

^1^ food additive other than phenolic compound (in this column, the brackets indicate: equal (0), increased (+) or decreased (-) antimicrobial capacity of food additives compared to phenolic compounds), ^2^ references, * application of phenolic treatment in packaging; O: oil, E: extract, P: powder, TVC: total viable count, LAB: lactic acid bacteria, BHT: butylated hydroxytoluene, CH: chitosan, TC: α-tocopherol, SN: sodium nitrite, AC: acetic acid, LA: lactic acid, BHA: butylated hydroxyanisole, CO: cochineal, SA: sodium ascorbate, DMSO: dimethylsulfoxide, NLD: nitrite, lactate and diacetate.
